# Neuronavigator-guided ventriculoscopic approach for symptomatic xanthogranuloma of the choroid plexus in the lateral ventricle

**DOI:** 10.1097/MD.0000000000014718

**Published:** 2019-04-19

**Authors:** Chengrui Yan, Shan Zhu, Haitao Sun, Wan-Ting Lee (Jenn), Xiaoying Zhang, Zongsheng Xu, Xiangyi Kong, Xiaolin Chen

**Affiliations:** a Department of Neurosurgery, Peking University International Hospital; b Department of Obstetrics and Gynecology, Peking Union Medical Collage Hospital, Chinese Academy of Medical Sciences; c Department of Anesthesiology, National Cancer Center/National Clinical Research Center for Cancer/Cancer Hospital, Chinese Academy of Medical Sciences and Peking Union Medical College, Beijing, China; d Mater Hospital Brisbane Queensland Medical Program, The University of Queensland, Brisbane, Australia; e Department of Pathology, Peking University International Hospital; f Department of Breast Surgical Oncology, National Cancer Center/National Clinical Research Center for Cancer/Cancer Hospital, Chinese Academy of Medical Sciences and Peking Union Medical College; g Department of Neurosurgery, Beijing Tiantan Hospital, Capital Medical University, Beijing, China.

**Keywords:** lateral ventricle, symptomatic xanthogranulomas of choroid plexus, ventriculoscopic approach

## Abstract

Xanthogranuloma of choroid plexus is an extremely rare, benign, and mostly asymptomatic intracranial lesion. We report a case of symptomatic lateral ventricular xanthogranuloma resected via a neuronavigator-guided ventriculoscopic approach. Then we review recent English medical literature and notice that craniotomies have been the most popular treatment. But our choice of a ventriculoscopic approach possesses unique advantages such as minimized neural tissue damage, shortened operative time, less blood loss, and safer access to central structures over conventional open surgeries. Informed consent has been obtained from the patient and his immediate family regarding this case report.

## 1 Introduction

Xanthogranulomas (XGs) of choroid plexus are rare, silent, and benign lesions of obscure origin in central nervous system with an incidence of 1.6% to 7.0% in autopsies.^[1,2]^ They rarely cause neurologic dysfunctions only when the neoplasms obstruct cerebrospinal fluid (CSF) pathway. In those symptomatic cases, surgical excision is the most common choice of treatment. To our knowledge, among all symptomatic lateral ventricular choroid plexus XGs, only 16 cases have been reported in English-language articles to date,^[3–18]^ with 14 patients treated by conventional craniotomy, another 2 underwent radiosurgeries^[15]^ or laser interstitial thermal therapy (LITT).^[18]^ Here we present another symptomatic case of XG of the choroid plexus located in the lateral ventricle, which has been innovatively resected through a ventriculoscopic approach. We also review recent literature concerning different therapies for such lesions.

## 2 Case report

### 2.1 Presentations and examinations

A 31-year-old man complained of a chronic slight bilateral headache for more than 10 years. He was admitted to hospital because the symptom had been progressive within 1 year. He denied any occurrences of fever, nausea, vomiting, altered consciousness, sensory or movement disorder, visual disturbances, facial palsy, aphasia, incontinence, or convulsion.

**Figure 1:**
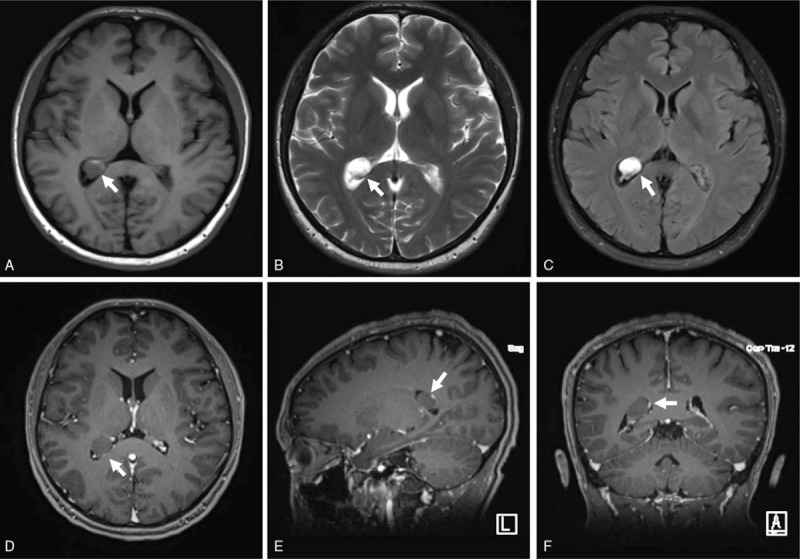
Preoperative magnetic resonance imaging showing a 1.3 × 1.2 × 1.0 cm oval mass filling the dilated posterior horn of the right lateral ventricle (white arrows). The lesion appeared hypointense on T1WI (A) including some hyperintense spots, and hyperintense on T2WI (B) as well as FLAIR images (C). Following contrast administration, no obvious enhancement was observed (D–F).

Vital signs were stable and neurologic examinations were normal. Results of laboratory examinations were unremarkable. Magnetic resonance imaging (MRI) demonstrated a dilated posterior horn of the right lateral ventricle filled with a well-delineated oval mass, measuring 1.3 × 1.2 × 1.0 cm (Fig. 1). The lesion appeared hypointense on T1-weight images (T1WI, Fig. 1A), including some hyperintense spots, and hyperintense on T2-weight (T2WI, Fig. 1B) as well as fluid-attenuated inversion recovery (FLAIR) images (Fig. 1C). Following contrast administration, it did not show obvious enhancement (Fig. 1D–F).

### 2.2 Treatment

**Figure 2:**
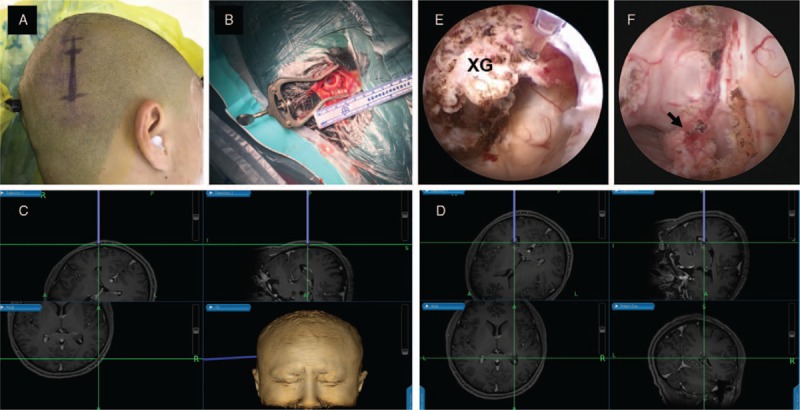
The patient's position, the scalp incision (A) and the burr hole (B). (C, D) A stereotactic navigation guidance system is used for a precise and straightforward trajectory to the lesion. (E) Structures of right lateral ventricle and the xanthogranuloma (XG) attached to the choroid plexus under endoscopic vision. (F) Choroid plexus after resection of XG in right lateral ventricle (black arrow).

After excluding all contraindications, a neurosurgery was performed on the patient. To minimize normal tissue destruction, we innovatively treated the patient through a stereotactic neuronavigator-guided ventriculoscopic procedure. The entrance point was carefully determined according to the best trajectory obtained from preoperative MRI. After general anesthesia and successful intubation, the patient was posed supinely with his head turned to the left side and fixed in a head holder (Fig. 2A). The patient's right scalp was prepared by 2% iodine together with 75% ethanol. Then a 3-cm incision (Fig. 2A) and a small burr hole (Fig. 2B) were made according to the surgical plan. The working sheath of the ventriculoscopy guided by a stereotactic navigation guidance system approached to the target point accurately and safely (Fig. 2C, D). After arriving at the target, clear structures of right lateral ventricle could be seen through the endoscopic vision, with a dark red, smooth-surfaced entity attached to the choroid plexus (Fig. 2E). The lesion was removed after bipolar coagulation, using microscissors, grasping forceps, and gentle aspiration. There was no bleeding in the surgical field and the trajectory under close inspection (Fig. 2F). The procedure was satisfactory, with approximate 50 mL blood loss.

### 2.3 Postoperative course

**Figure 3:**
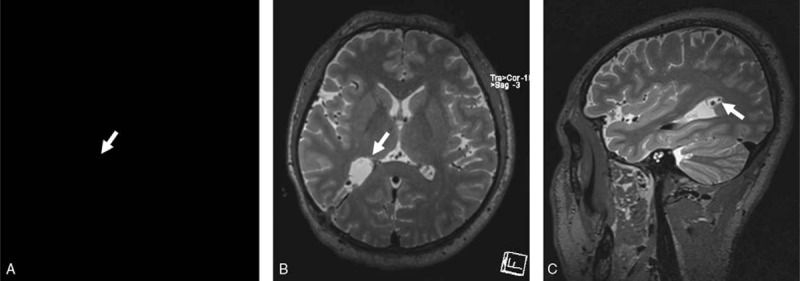
Postoperative magnetic resonance imaging showing no residual tumor (white arrows).

Postoperative MRI confirmed a gross total resection of the tumor (Fig. 3). The patient recovered uneventfully and was discharged on the 7th postoperative day. He reported a slight but much better headache at the 1-month follow-up visit.

### 2.4 Histologic examination

**Figure 4:**
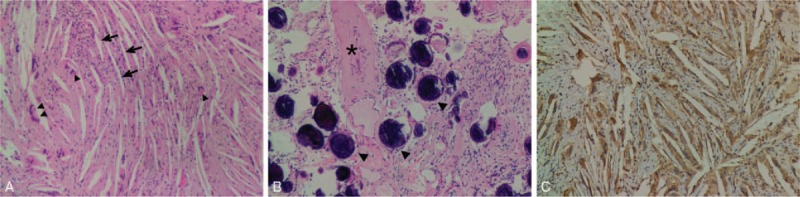
Photomicrographs of the surgical specimen. (A) Typical cholesterol clefts (arrows) and granulomas (arrowheads) enclosed by inflammatory cells (hematoxylin and eosin [H&E] stain, ×100). (B) Hyalinized and calcified blood vessels (asterisk) and psammoma bodies (arrowheads) (H&E stain, ×100). (C) Positive CD68 immunostaining in histiocytic cells surrounding cholesterol clefts and granulomas (H&E stain, ×100).

Microscopically, the surgical specimen was mainly composed of cholesterol crystals or clefts and granulomas, infiltrated by lymphocytes, plasma cells, and eosinophils. In some areas, there were several thick- or thin-walled blood vessels with hyalinization and calcification, as well as numerous psammoma bodies. Choroid plexus epithelium and focal areas of fibrous connective tissue were seen in the lesions. On immunohistochemistry, CD68 was positive in histiocytic cells and CD31 was positive in vessels. Glial fibrillary acidic protein was not detected. Ki-67 index was low, <1% (Fig. 4).

The final histopathologic diagnosis was a XG of choroid plexus.

## 3 Discussion

Xanthogranuloma in man was 1st described by Blumer in 1900 under the initial diagnosis of “cholesteatomatous endothelioma.” Later such lesions appeared in different reports on a variety of names such as “cholesterinhaltige Geschwulste,” ”xanthoma,” “cholesteatoma des plexus choroïdes,” “choléstéatome des plexus choroïdes,” ”cholesterol granuloma,”and “xanthogranuloma” (cited by Jaer et al).^[5]^ Ever since, they have been believed benign lesions and the vast majority of cases were incidental findings in postmortem examinations and their prevalence was estimated at 1.6% to 7.0% according to data from different studies.^[1,2]^ From Wolf et al^[1]^ and Ayres and Haymaker,^[19]^ it seemed that older individuals were more vulnerable than the younger and incidence in either sex had no difference. However, large scale studies are needed for a more representative conclusion. Pathogenesis remained controversial. Shuangshoti et al suggested that due to proved proliferative capacity of the choroid plexus epithelium, epithelial cells could desquamate from the surface, enter and degenerate in the stroma of the plexus via sites of disruption of the basement membrane. As a result, lipid would accumulate in then being released from these cells which provoked a response from chronic inflammation.^[20,21]^ This theory was widely accepted because it explained the typical histological picture composed of clusters of xanthoma cells, cholesterol crystals or clefts, and fibrous proliferation enclosed by inflammatory cells such as foreign-body giant cells, plasma cells, or lymphocytes. And occasionally, calcium deposits, focal hemorrhage, or cyst formation to the original lesions could be seen.^[1,22,23]^ There were rare but still some XGs of the choroid plexus that could produce symptoms especially when they obstructed normal CSF flow or compressed structures nearby. Patients could complain of headache, hemiparesis, gait disturbance, hemiparesis, seizure, memory deterioration, parosmia, parageusia (presented in Table 1) and even sudden death caused by acute and severe hemorrhage.^[23]^ CT and MRI were helpful in detecting such lesions, but their diagnostic value was limited. On CT image, the masses varied from hyper- to hypodense compared to normal brain tissues. They usually appeared iso- or hyperintense on T1WI, hyperintense on T2WI and FLAIR images, with associated brain edema presented sometimes. And they could be slightly or partially enhanced after contrast administration.^[24]^

**Table 1:**
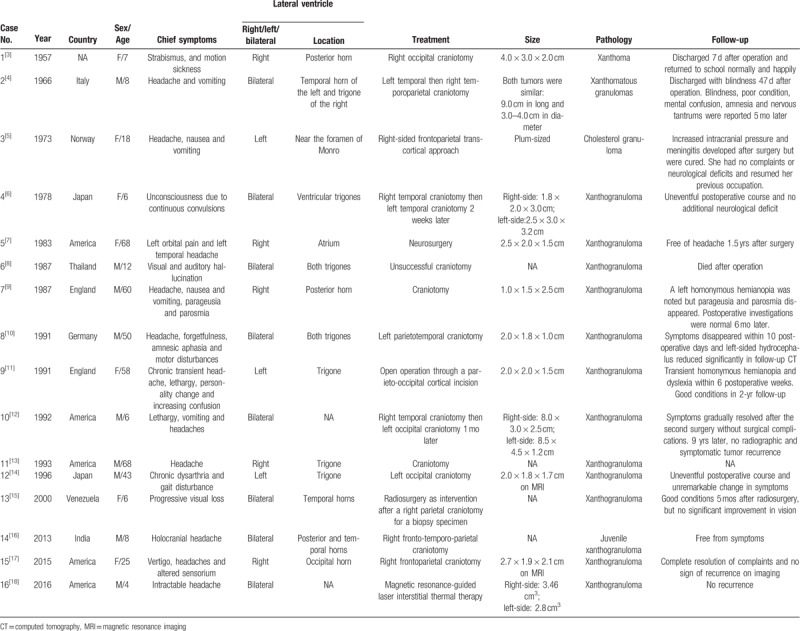
Clinicopathologic features of the included studies.

Every symptomatic case required medical interventions, ablation, or resection, to prevent deterioration of conditions. For a further insight of various surgical procedures, we have reviewed recent literature published in English. On-line databases such as PubMed, Embase, Chinese National Knowledge Infrastructure (CNKI), and Wanfang were searched for potential articles updated to January 4, 2018. And finally, a total of 16 articles reporting 16 cases of symptomatic lateral ventricular choroid plexus XGs were selected, excluding those located at the foramen of Monro. Clinicopathologic features of the included 16 patients have been are in Table 1. Fourteen of 16 patients received open surgeries and another 2 were treated by radiosurgery or magnetic resonance-guided laser interstitial thermal therapy. All the surgical procedures were craniotomies through different approaches determined by mass locations. Surgical results were generally good except for 1 postoperative death.^[8]^ Eeven patients^[3–7,9–12,16,17]^ demonstrated remarkable improvement or even complete remission of their clinical status but 1 patient had no change in primary symptoms.^[14]^ There was 1 report that provided no information on postoperative course.^[13]^ The main complications included increased intracranial pressure and meningitis in 1 patient,^[5]^ sudden persistent blindness in 1 patient,^[4]^ transient hemianopia in 2 patients,^[9,11]^ and dyslexia in 1 patient.^[11]^ To our knowledge, we report the world 1st case of symptomatic lateral ventricular choroid plexus XG that was resected via a ventriculoscopic approach instead of an open surgery. The well-established stereotactic navigation guidance system helped to determine the best trajectory. The patient recovered more quickly and uneventfully from surgical stress without any complication.

As shown in Table 1, craniotomies via transcallosal and transcortical–transventricular approaches to the lateral ventricle have been the most common choice for the removal of intraventricular XG. But risk of surgical complications mentioned above was inevitable due to extensive damage to neural tissue. In recent years, there have been a growing number of studies proposing that ventriculoscopes were more effective and less traumatic when applied to remove benign intraventricular cystic lesions.^[25–28]^ Revolutions in endoscopes and instruments specifically designed for microneurosurgery and routine use of neuronavigation have improved accuracy of ventriculoscopic surgeries and reduced operative damages. Inspired by these facts and our rich experience in ventriculoscopy, we tried to apply such comprehensive techniques to lateral ventricular XG resection in this case then achieved good results. In our review of the literature, we did not find any previous reports on this the modality for such entities.

In our opinion, although neuronavigation-guided ventriculoscopic procedures are risky, there are advantages of them over the conventional open craniotomies as follows: minimized neural tissue transection and negligible cranial nerve manipulation, excellent intraventricular picture to avoid damage of critical structures, shortened operative time and less blood loss, and safer access to central structures that require extensive or difficult dissection such as foramen of Monro and forth ventricle. Nevertheless, one of the restrictions of such procedures is the fact that they are quite technically demanding. And there must be some cases in which radical resection is extremely difficult. Moreover, the patient's follow-up examinations remain to be done so that we could evaluate the long-term prognosis.

We believe that, as one of minimally invasive neurosurgical procedures, neuronavigation-guided ventriculoscopy is a promising alternative to conventional open surgeries for symptomatic XG of the lateral ventricular choroid plexus and other benign lateral ventricular entities.

## 4 Conclusion

We have presented our initial experience of successfully resecting a XG of the choroid plexus in the lateral ventricle through a neuronavigator-guided ventriculoscopic approach. Our review of literature suggested that it was an innovation in surgical treatment of symptomatic lateral ventricular XGs with unique advantages of minimized neural tissue damage, shortened operative time, less blood loss, and safer access to central structures.

## Author contributions

**Conceptualization:** Xiangyi Kong, Chengrui Yan, Shan Zhu, Zongsheng Xu, Haitao Sun, Wan-Ting Lee.

**Data curation:** Xiaoying Zhang.

**Formal analysis:** Xiaolin Chen.

**Writing – review & editing:** Xiangyi Kong.
